# Forcing contacts between mitochondria and the endoplasmic reticulum extends lifespan in a *Drosophila* model of Alzheimer's disease

**DOI:** 10.1242/bio.047530

**Published:** 2020-01-14

**Authors:** Juan Garrido-Maraver, Samantha H. Y. Loh, L. Miguel Martins

**Affiliations:** MRC Toxicology Unit, University of Cambridge, Lancaster Road, Leicester LE1 9HN, UK

**Keywords:** *Drosophila*, Organelle contacts, Mitochondria, Endoplasmic reticulum, Alzheimer's disease

## Abstract

Eukaryotic cells are complex systems containing internal compartments with specialised functions. Among these compartments, the endoplasmic reticulum (ER) plays a major role in processing proteins for modification and delivery to other organelles, whereas mitochondria generate energy in the form of ATP. Mitochondria and the ER form physical interactions, defined as mitochondria–ER contact sites (MERCs) to exchange metabolites such as calcium ions (Ca^2+^) and lipids. Sites of contact between mitochondria and the ER can regulate biological processes such as ATP generation and mitochondrial division. The interactions between mitochondria and the ER are dynamic and respond to the metabolic state of cells. Changes in MERCs have been linked to metabolic pathologies such as diabetes, neurodegenerative diseases and sleep disruption. Here we explored the consequences of increasing contacts between mitochondria and the ER in flies using a synthetic linker. We showed that enhancing MERCs increases locomotion and extends lifespan. We also showed that, in a *Drosophila* model of Alzheimer's disease linked to toxic amyloid beta (Aβ), linker expression can suppress motor impairment and extend lifespan. We conclude that strategies for increasing contacts between mitochondria and the ER may improve symptoms of diseases associated with mitochondria dysfunction. A video abstract for this article is available at https://youtu.be/_YWA4oKZkes.

This article has an associated First Person interview with the first author of the paper.

## Abstract (precis)

**Summary:** Enhancing mitochondria–ER contacts ameliorates locomotor phenotypes and extends lifespan in a fly model of Alzheimer's disease.

## INTRODUCTION

Eukaryotes include several unicellular and multicellular organisms, including fungi, plants and animals. Eukaryotes differ from prokaryotes in that they have a compartmentalised cytoplasm with membrane-bound organelles. Organelles give eukaryotic cells several advantages, including providing special environments that favour specific biochemical reactions required for life. In eukaryotic cells, the endoplasmic reticulum (ER) provides an environment for protein and phospholipid synthesis and the storage of ions such as Ca^2+^. Mitochondria are responsible for the conversion of energy stored in the chemical bonds of nutrients into ATP, the cellular energy currency. Organelles have been studied in isolation, but now it is clear that they also form contacts. The most well-characterised organelle contact sites are formed between the ER and mitochondria (reviewed in Rowland and Voeltz, 2012). Sites of contact between mitochondria and the ER regulate lipid synthesis, Ca^2+^ signalling, mitochondrial energy generation and mitochondrial division.

Alterations in contacts between mitochondria and the ER can cause mitochondrial calcium overload and an increase in mitochondrial reactive oxygen species (ROS), and such alterations in the liver have been linked to obesity, a medical condition, in mice (Arruda et al., 2014). In neurons, alterations in contacts between mitochondria and the ER can result in neuronal cell death (Celardo et al., 2016), defects in the release of synaptic vesicles (Valadas et al., 2018) and ER stress (Celardo et al., 2016; Schneeberger et al., 2013).

The connections between mitochondria and the ER have been manipulated in cultured cells and increasing such links can commit cells to a death pathway (Csordás et al., 2006). Synthetic linkers that increase contacts between the ER and mitochondria in the mouse liver induce a transient induction of ER stress that resolves over time (Arruda et al., 2014).

Here, we report a *Drosophila* model for expressing a synthetic linker between the mitochondria and the ER. We used this *in vivo* model to explore the long-term consequences of forcing contacts between these two organelles. We showed that flies in which the contacts between mitochondria and the ER are enhanced have a fragmented mitochondrial network and increased levels of ROS. The sustained expression of an artificial linker also leads to enhanced motor activity and extended lifespan. We also showed that the improved health span conferred by the expression of this artificial linker counteracts the toxic effects resulted from the expression of a toxic version of amyloid-β (Aβ) which causes Alzheimer's disease in humans.

## RESULTS

### Tethering ER and mitochondria in *Drosophila* using an engineered linker

The association between mitochondria and the ER has been widely documented in different experimental models. The study of this association has been the focus of intensive research, using both cellular and animal models. However, the diversity of the reported functions of these contacts suggests that their role is still unclear (reviewed in Csordás et al., 2018).

**Fig. 1.**
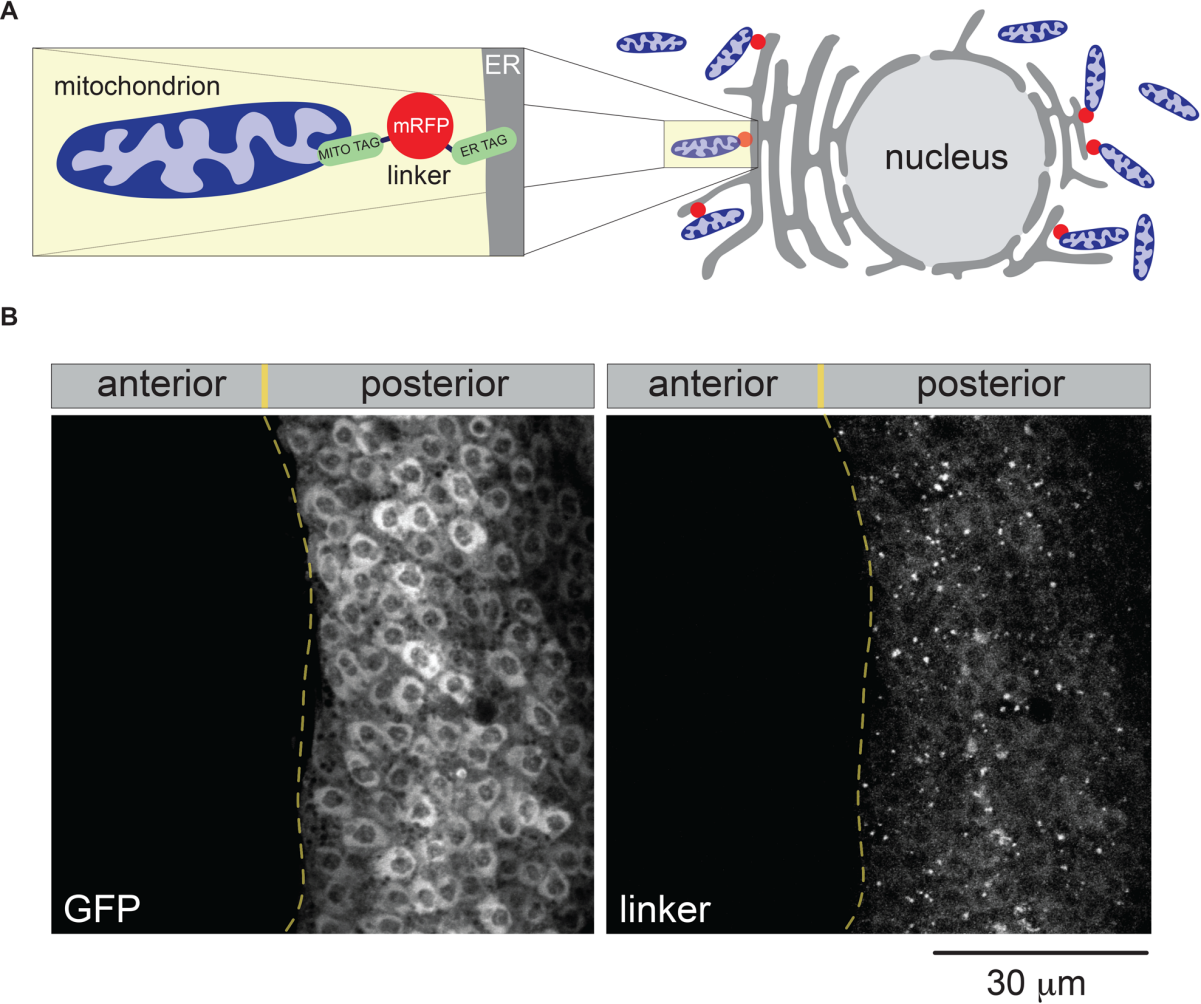
***In vivo* expression analysis of an engineered tether between mitochondria and the ER.** (A) A schematic illustration of the linker tethering mitochondria (blue) and the ER (grey). The magnified panel on the left details the relative position of the two tags (green) targeting both mitochondria and the ER and bridged by monomeric RFP. (B) Analysis of the expression of the linker targeted to the posterior compartment of the larval wing disc. Genotype: *w; UAS-linker/+; hhGal4, UAS-GFP/+*.

Here, we used a genetic approach to explore the effects of tethering the ER and mitochondria in fruit flies (*Drosophila melanogaster*) using an artificial linker. To increase the physical coupling between both organelles, we used a previously reported linker that induces enhanced proximity (6 nm) between mitochondria and the ER in mammals (Csordás et al., 2006). We first engineered a codon-optimised version of this synthetic linker for expression in *Drosophila*. This synthetic linker (from here on referred to as ‘linker’) consists of monomeric red fluorescent protein (RFP) with a mitochondrial targeting sequence at the N terminus and an ER targeting sequence at the C terminus (Fig. 1A). Transgenic flies expressing this construct were generated by PhiC31 integrase-mediated transgenesis (see Materials and Methods) and the expression of the linker was assayed by immunofluorescence. Confocal analysis of the larval wing discs revealed punctate staining of the linker (Fig. 1B).

### Linker expression increases MERCs and decreases mitochondrial length

**Fig. 2.**
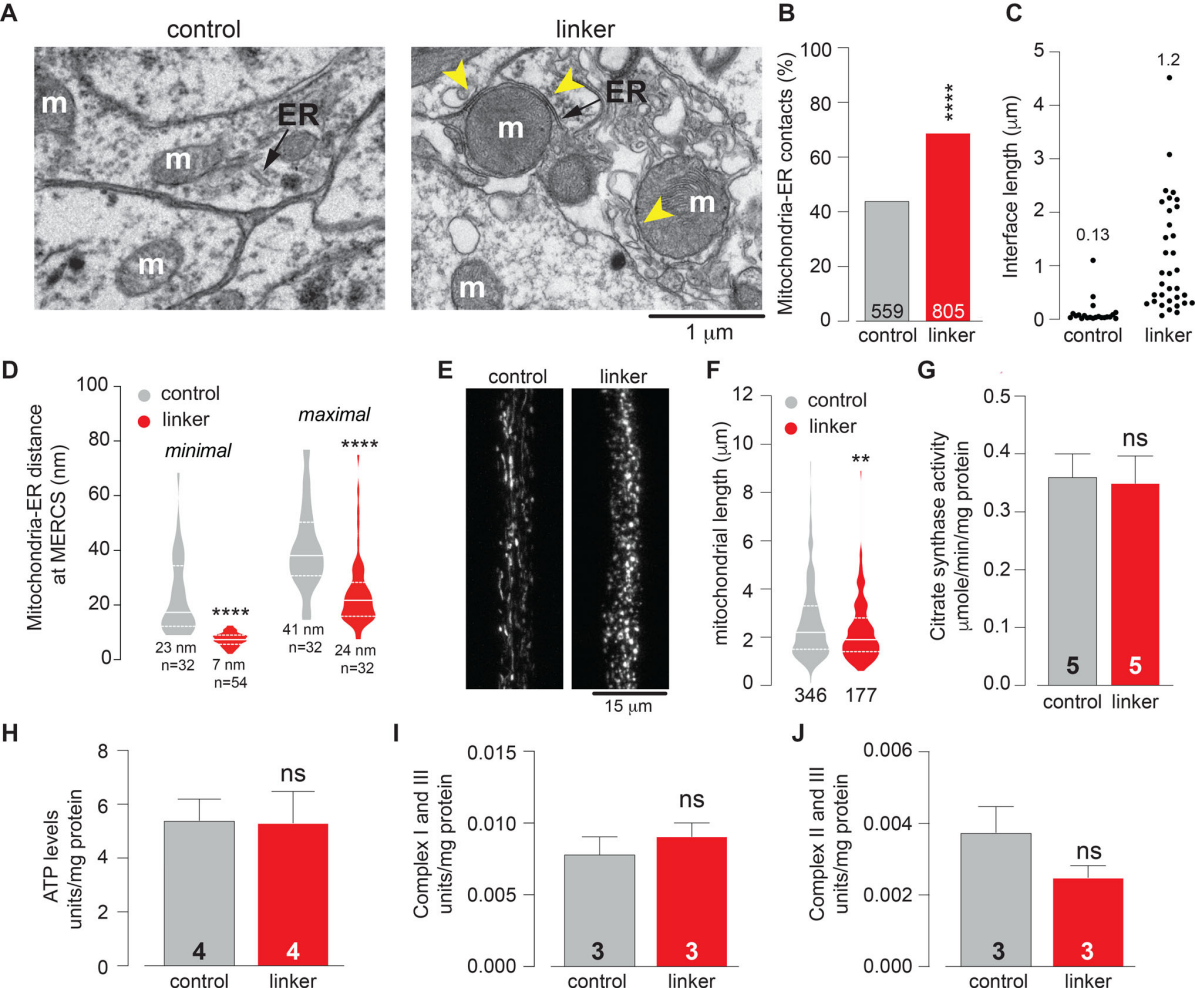
**Forcing contacts between mitochondria and the ER induces mitochondrial fragmentation.** (A,B) The quantification of mitochondria–ER contacts in adult fly brains (asterisks, chi-square two-tailed, 95% confidence intervals) (B) and representative electron microscopy images (A). The yellow arrows show mitochondria in contact with the ER (arrows). ER, endoplasmic reticulum; m, mitochondria. (C) Analysis of the length of the interface between mitochondria and the ER in adult brains. The mean is shown above the individual measurements. (D) Analysis of the distance between mitochondria and the ER at MERCs. Both the minimum and maximum observed distance at each individual MERC was measured. The quantification of distance is shown as a combined violin and box plot. The mean distance is also shown. *P*-value, two-tailed unpaired *t*-test, compared to control. (E,F) The expression of the linker results in mitochondrial fragmentation. Confocal analysis of mitoGFP in the larval mechanosensory axons. Representative confocal images (E) and the quantification of mitochondrial length (F). The quantification of mitochondrial length is shown as a combined violin and box plot (*P*-value, two-tailed unpaired *t*-test, compared to control). (G) The expression of the linker does not affect overall mitochondrial mass. Mitochondrial mass assessed by measuring the activity of the mitochondrial matrix enzyme citrate synthase in adults (mean±s.d., ns, *P* > 0.05, two-tailed unpaired *t*-test, compared to control). (H–J) The expression of the linker does not alter ATP levels (H) or mitochondrial function (I,J). Mitochondrial function was assayed by measuring the activity of NADH: cytochrome c reductase (I) and succinate: cytochrome c reductase (J) (mean±s.d., ns, *P* > 0.05, two-tailed unpaired *t*-test, compared to control). Genotypes (A–D): control: w; elavGal4/+; +, linker: w; elavGal4/UAS-linker; +, (E,F): control: w; elavGal4/+; UAS-mitoGFP/+, linker: w; elavGal4/UAS-linker; UAS-mitoGFP/+, (G–J): control: w; +; daGal4/+, linker: w; UAS-linker/+; daGal4/+.

**Fig. 3.**
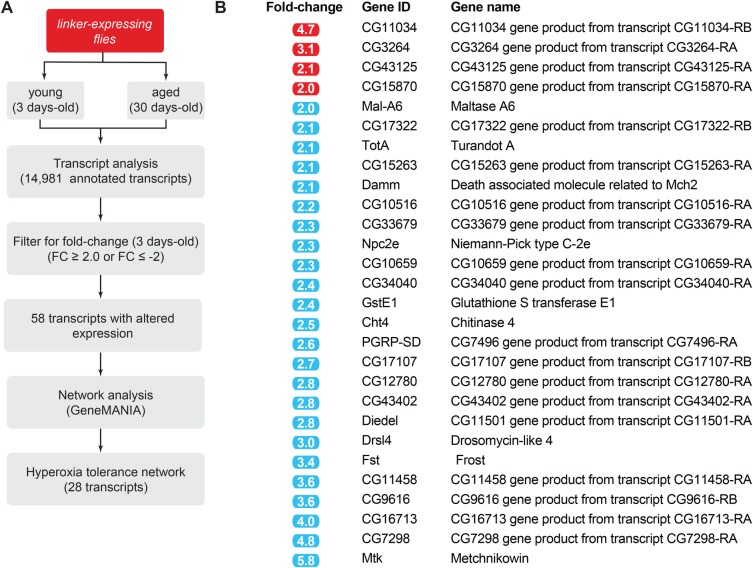
**A transcriptional signature associated to high oxygen tolerance is induced by enhancing MERCs.** (A) Workflow used for the characterisation of transcripts involved in tolerance to hyperoxia in adult flies expressing the linker. (B) Transcripts with altered expression in flies expressing the linker. Red and blue correspond to significant (FDR ≤ 5%) upregulated and downregulated transcripts, respectively. The original signature for hyperoxia tolerance transcripts was identified by Zhao and colleagues (Zhao et al., 2010).

Next, to determine if linker expression increases mitochondria–ER tethering, we quantified mitochondria–ER contacts in the adult brains as previously described (Celardo et al., 2016). Ultrastructural analysis by transmission electron microscopy (TEM) revealed a significant increase in mitochondria–ER contacts in flies expressing the linker compared to controls (Fig. 2A and B). We next compared the length of the interface between mitochondria and the ER and the distance between the two organelles in flies expressing the linker to the controls. This showed that linker expression increases the average length of the interface (Fig. 2C) and decreases the distance between mitochondria and the ER (Fig. 2D). The points of contact between the ER and mitochondria correlate with the localisation of dynamin-related protein 1 (DRP1), a protein involved in mitochondrial fission in vertebrate cells. Such contact sites have been proposed to be involved in mitochondrial division (reviewed in Rowland and Voeltz, 2012). Confocal analysis of mitochondria labelled with a fluorescent tag (mito-GFP) in the mechanosensory neurons of the ventral nerve cord showed that the expression of the linker resulted in decreased mitochondrial length (Fig. 2E and F). Mitochondrial fragmentation in *Drosophila* has been proposed to drive the removal of defective mitochondria by mitophagy (Lieber et al., 2019). To determine whether linker expression affects mitochondrial removal through fragmentation, we next assessed mitochondrial density by measuring the levels of the mitochondrial matrix enzyme citrate synthase, an indirect measure of mitochondrial density in flies (Magwere et al., 2006). Citrate synthase levels in flies expressing the linker were not altered compared to those in controls (Fig. 2G), indicating that the mitochondrial fragmentation observed upon linker expression was not accompanied by a loss of mitochondrial mass. Mitochondria generate the majority of ATP as a source of cellular energy. We measured total levels of ATP in adult flies expressing the linker but did not find significant changes in ATP levels compared to those in controls (Fig. 2H). We also assessed the functional status of mitochondria by measuring the activity of respiratory complexes in flies expressing the linker and found no significant changes (Fig. 2I and J). Together, these results show that forcing contacts between mitochondria and the ER using an artificial linker induces mitochondrial fragmentation without altering global mitochondrial density or activity. Enhancing MERCs led to a fragmented mitochondrial network. In flies this fragmentation is associated with the transcriptional activation of the mitochondrial unfolded protein response (UPR^mt^) (de Castro et al., 2012) that can enhance lifespan (Jensen et al., 2017). To determine if enhancing MERCs is associated with a transcriptional stress response, we employed microarray technology with an *in silico* workflow (Fig. 3A). We failed to detect alterations in transcripts associated with mitochondrial fission or fusion (data not shown) but instead identified transcriptional alterations associated with resistance to high oxygen environments in flies (Fig. 3B).

### Enhancing MERCs causes increased locomotor activity

**Fig. 4.**
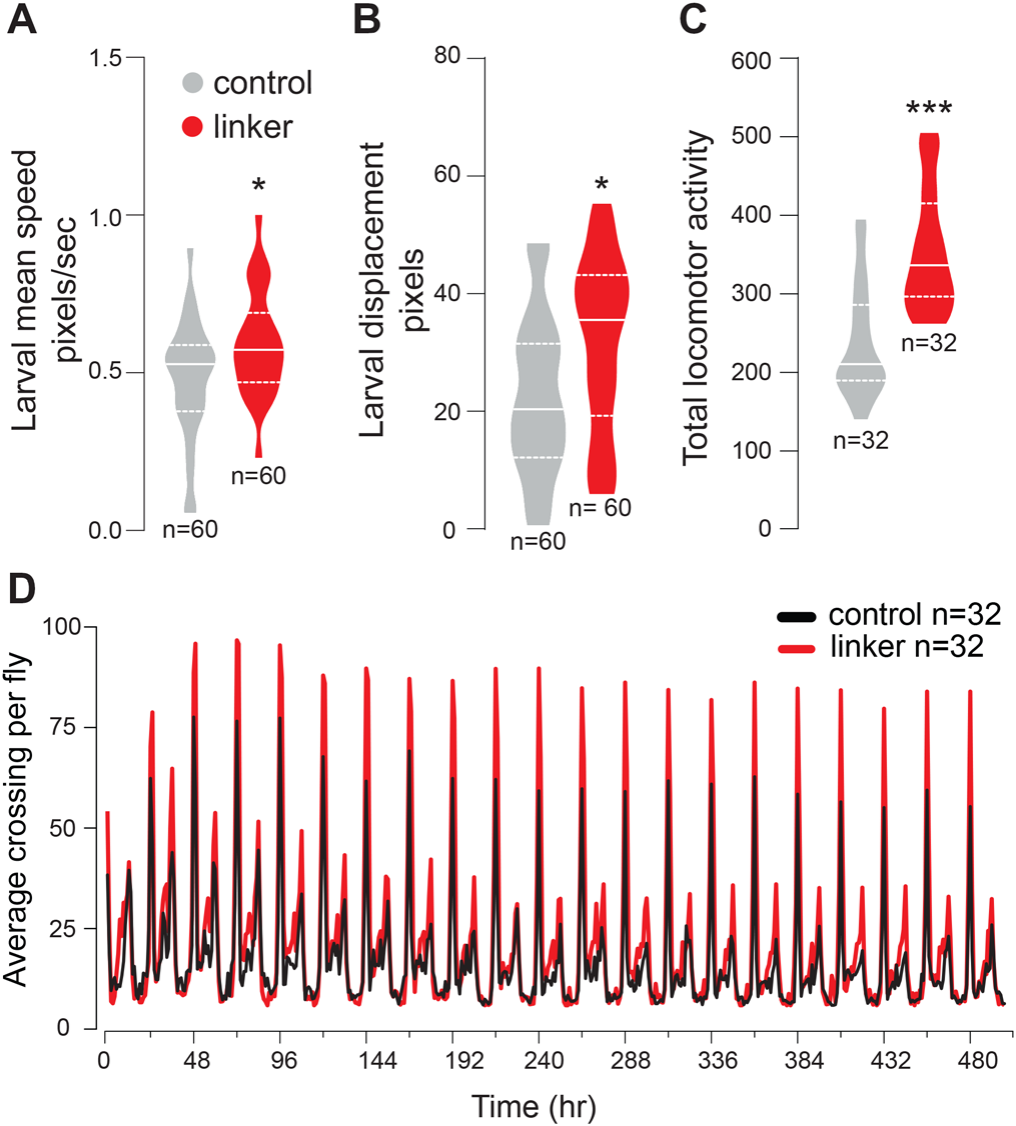
**Enhancing MERCs increases locomotor activity.** (A,B) Analysis of larval crawling in control and linker-expressing animals. Larval mean speed (A) and displacement (B) were measured (mean±s.d.; asterisk, two-tailed unpaired *t*-test). (C) Total activity in adult flies over a period of 20 days was measured using the Trikinetics system. (mean±s.d.; asterisks, two-tailed unpaired *t*-test compared to control). (D) Actogram showing that the linker expressing flies have a higher average activity per hour than control over a period of 480 h. Genotypes: control: *w; +; daGal4/+*, linker: *w; UAS-linker/+; daGal4/+.*

We have previously shown that increased mitochondrial fission in flies with decreased expression of *Drosophila* mitofusin (*dMfn*) causes locomotor defects in adults flies (Garrido-Maraver et al., 2019). To determine whether mitochondrial fission caused by linker expression affects motor activity, we measured locomotion in both larvae and adults by expressing the synthetic linker ubiquitously. First, we analysed locomotor activity in larvae using a motility tracking assay. We found a significant increase in the crawling speed (Fig. 4A) and total displacement (Fig. 4B) of larvae expressing the linker. Next, analysis of locomotor activity in adult flies for a period of up to 20 days showed a generalised increase in the activity of flies expressing the linker (Fig. 4C and D).

### Calcium levels are increased at mitochondria–ER contacts

**Fig. 5.**
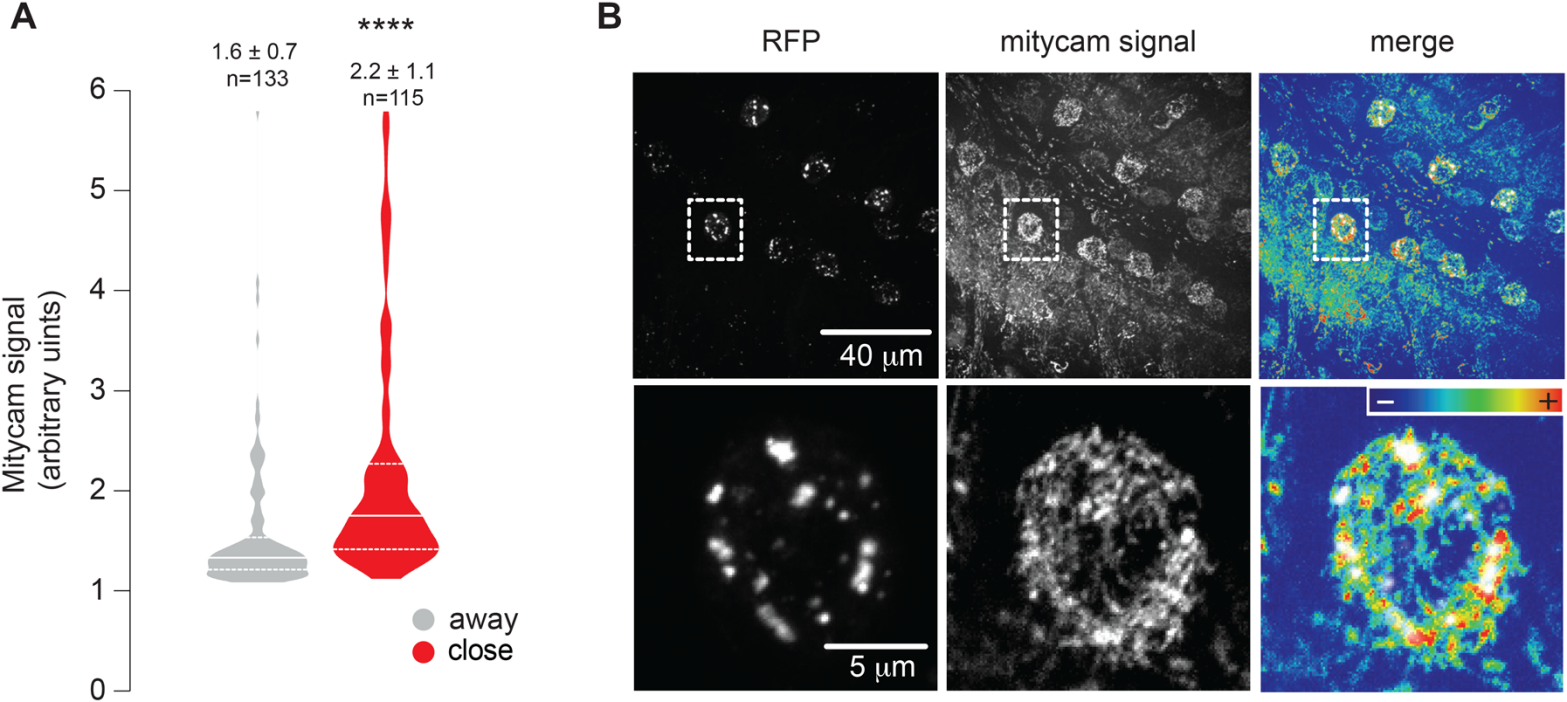
**Increased calcium levels in mitochondria at MERCs.** (A,B) Analysis of mitochondrial calcium uptake in neurons at the ventral nerve cord in larvae expressing the linker. The signal of the mitycam reporter (mitycam signal) was analysed close to and away from the linker signal (RFP). (A) The quantification of the mitycam fluorescence [mean±s.d.; asterisks, two-tailed unpaired *t*-test compared to control, *n*=region of interest (ROI) measured]. (B) Representative confocal images of the mitycam signal and the linker in larval brain cells. Genotypes (A,B): *w; elavGal4,UAS-mitycam/ UAS-linker*.

MERCs play a key regulatory role in several cellular functions, including the transfer of Ca^2+^ and lipids between the ER and the mitochondria (reviewed in Rowland and Voeltz, 2012). Additionally, in hepatocytes, Ca^2+^ transport from the ER to mitochondria at contact sites elevates mitochondrial Ca^2+^ and increases mitochondrial ROS (Arruda et al., 2014). To measure Ca^2+^ levels in the mitochondria of flies expressing the linker, we used mitycam, a mitochondria-targeted fluorescent calcium reporter for *Drosophila* (Terhzaz et al., 2006). As our artificial linker is tagged with RFP, we compared the mitycam signal in areas of RFP fluorescence (which correspond to mitochondria–ER contacts) to that in areas in which no RFP fluorescence was detected (which correspond to mitochondria distant from the ER). This revealed that mitochondria close to the ER contain higher levels of Ca^2+^ when compared to those in mitochondria distant from the ER (Fig. 5A and B).

### Artificially increasing MERCs increases ROS levels and enhances longevity

**Fig. 6.**
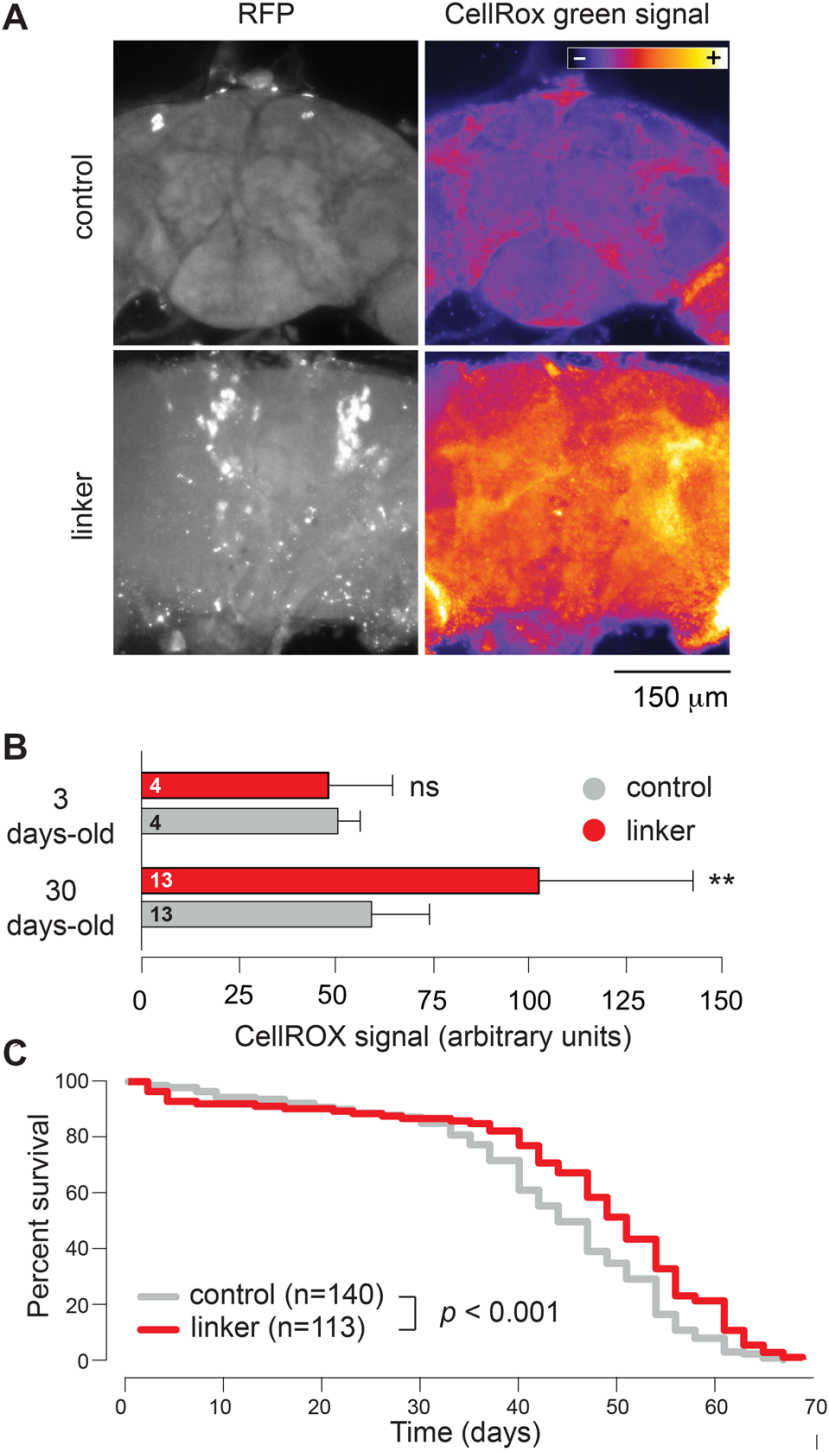
**Enhancing MERCs increases ROS levels in aged flies and increases lifespan.** (A,B) Analysis of mitochondrial ROS levels in young (3-day-old) and aged (30-day-old) controls and linker-expressing flies. Representative confocal images of 30-day-old brains stained with CellRox green are shown (A). The quantification of CellRox green signal (mean±s.d., asterisks, two-tailed unpaired *t*-test, compared to control) (B). (C) Lifespan analysis was performed over a period of 70 days (asterisks, log-rank Mantel-Cox test). Genotypes (A–C): control: *w; +; daGal4/+*, linker: *w; UAS-linker/+; daGal4/+*.

We next tested whether the linker expression affects the levels of mitochondrial ROS in adult flies. We performed this analysis using CellRox, a fluorescent ROS indicator. Our analysis showed an age-dependent increase in the levels of ROS (Fig. 6A and B) in adult flies expressing the linker. It has been shown that increased ROS production in flies extends lifespan (Scialò et al., 2016). We therefore asked whether the effects on ROS levels induced by linker expression can affect the lifespan of adult flies. Our analysis showed that enhancing MERCs through linker expression extends lifespan (Fig. 6C).

### Increasing contacts alleviates alterations in locomotion and lifespan in flies expressing *Αβ_42_ arc*

**Fig. 7.**
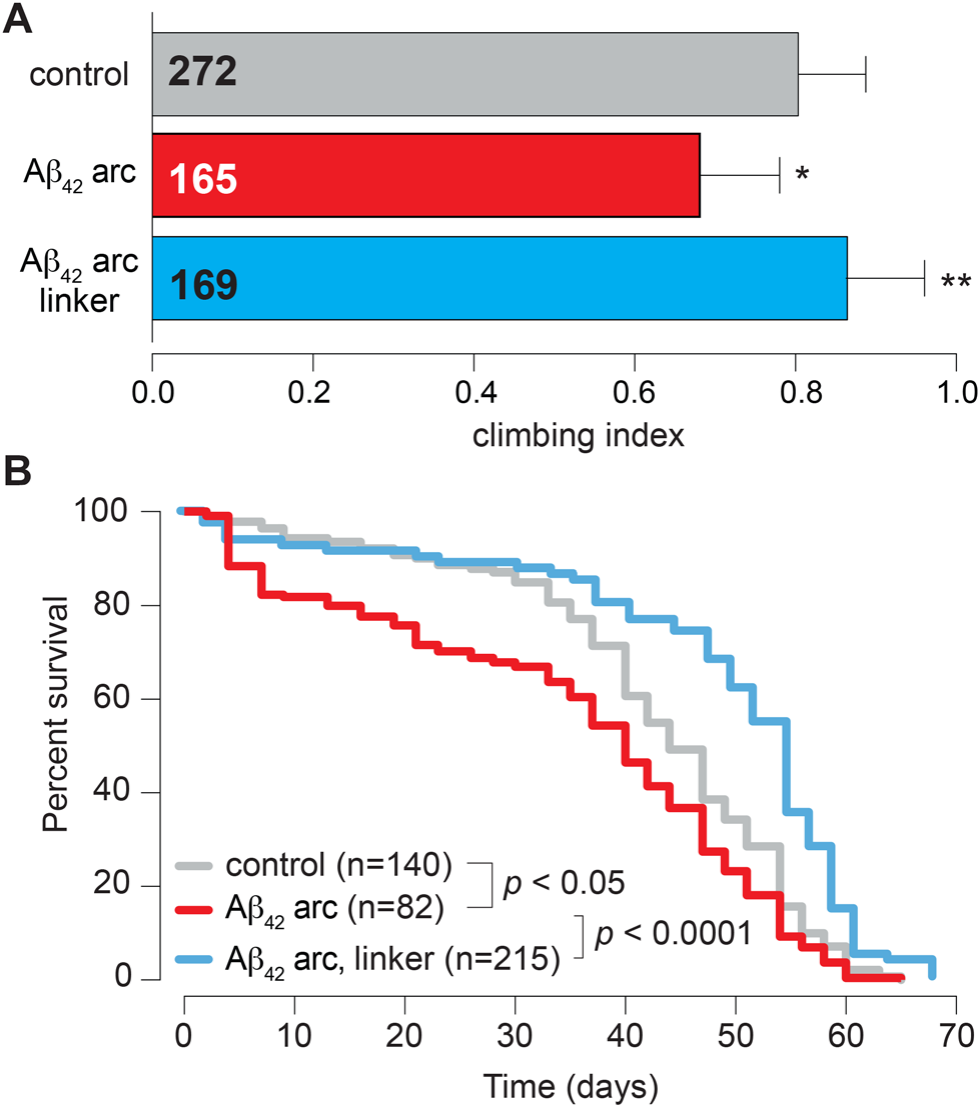
**Linker expression rescues locomotor defects and increases lifespan in flies expressing toxic amyloid beta.** (A) Analysis of locomotion in controls, *Αβ_42_ arc flies* and *Αβ_42_ arc flies* expressing the linker. Flies were tested using a standard climbing assay (mean±s.d., asterisks, one-way ANOVA). (B) Lifespan analysis was performed over a period of 70 days in controls, *Αβ_42_ arc* flies and *Αβ_42_ arc flies* expressing the linker (asterisks, log-rank Mantel-Cox test). Genotypes (A,B): control: *w; +; daGal4/+*, *Αβ_42_ arc: w; +; daGal4/UAS-Αβ_42_ arc* and *Αβ_42_ arc, linker: w; UAS-linker/+; daGal4/UAS-Αβ_42_ arc*.

Signalling at mitochondria–ER contact points has been linked to several neurodegenerative disorders including Alzheimer's disease (AD) (Schon and Area-Gomez, 2013). In AD, an abnormal toxic protein in the brain, amyloid-β (Aβ), accumulates and causes neuronal cell death. *Drosophila* can be used to model AD through the expression of arctic mutant (Glu22Gly) Aβ peptides (*Αβ_42_* arc) in fly neurons. This causes progressive locomotor defects and the premature death of the flies (Crowther et al., 2005). We therefore tested whether forcing mitochondria–ER contacts by linker expression affects the toxic consequences of the expression of *Αβ_42_* arc in flies. We observed that linker expression suppressed climbing defects in flies expressing *Αβ_42_* arc (Fig. 7A). Lifespan analysis confirmed that the decreased lifespan of flies expressing *Αβ_42_* arc was suppressed by linker expression (Fig. 7B). We conclude that forcing mitochondria–ER contacts by the expression of the artificial linker confers protection in this *Drosophila* model of AD.

## DISCUSSION

In this study, we show that linker expression leads to a decrease in mitochondrial length (Fig. 2E and F). The ER controls mitochondrial biogenesis, a process that involves mitochondrial division. The division of mitochondria through fission occurs at MERCs, in which the division machinery is recruited and mitochondrial constriction occurs (reviewed in Rowland and Voeltz, 2012). It is therefore possible that the decreased mitochondrial length observed in flies expressing the linker is a consequence of increased levels of mitochondrial fission resulting from a higher number of MERCs.

We detected increased levels of Ca^2+^ in mitochondria in close proximity to the linker (Fig. 5A and B). Increases in Ca^2+^ levels in the mitochondrial matrix activate enzymes involved in the citric acid cycle, including pyruvate, isocitrate and α-ketoglutarate dehydrogenases (Denton and McCormack, 1985; Hansford, 1985) and stimulate oxidative phosphorylation, leading to an increased production of ATP. On the other hand, sustained levels of high Ca^2+^ in mitochondria are detrimental to their function (reviewed in Rizzuto et al., 2012). In this study, we did not measure the total levels of cellular Ca^2+^. We therefore cannot rule out that overall changes in intracellular stores are linked to the observed increase in mitochondrial Ca^2+^ in close proximity to the linker. We did not observe any global alterations in ATP levels (Fig. 2H) or in mitochondrial function (Fig. 2I and J) upon linker expression. However, given that we measured ATP and mitochondrial function globally and not in the subset of mitochondria in close proximity to the ER, it is possible that local effects were diluted when the biochemical assays used in this study were performed.

Forcing mitochondria–ER contacts by linker expression in mice has been shown to lead to an increase in markers of ER stress and higher ROS levels. However, this has only been observed in animals subjected to overnutrition by a high fat diet (Arruda et al., 2014). Here we detected a time-dependent increase in ROS levels in flies expressing an artificial linker without a specific dietary intervention. Historically, mitochondrial ROS have been associated with negative outcomes such as premature ageing (Forster et al., 1996) and diseases such as Parkinson's disease (Morais et al., 2014). However, it has been shown that, in flies, increasing ROS production specifically through respiratory complex I reverse electron transport (RET) extends *Drosophila* lifespan (Scialò et al., 2016). Additionally, this RET-mediated effect of ROS is capable of enhancing the lifespan of flies with decreased expression of *pink1*, a gene linked to Parkinson's disease (Scialò et al., 2016). Here, we found that linker expression was associated with a gene expression signature linked to hyperoxia, higher ROS levels, enhanced longevity and the suppression of phenotypes associated with the expression of toxic Aβ. As toxic Aβ expression is linked to mitochondrial dysfunction (reviewed in Reddy and Beal, 2008), it would be important to determine if RET is the mechanism through which our artificial linker rescues the phenotype induced by toxic Aβ expression in flies. We propose that this could be investigated in future studies by epistasis analysis, for example, by expressing the alternative oxidase (AOX) of *Ciona intestinalis*, an enzyme that suppresses RET in flies (Scialò et al., 2016). If ROS-mediated RET is the mechanism through which linker expression rescues mitochondrial dysfunction in flies expressing toxic Aβ, AOX expression should block this rescue.

The optimal length for the transfer of Ca^2+^ between the ER and mitochondria is reported to range from 15–25 nm (reviewed in Giacomello and Pellegrini, 2016). Here, the expression of an artificial linker in flies increased the length of the interface between the two organelles by approximately ninefold (Fig. 2C) and decreased its maximal distance from an average of 41 nm to 24 nm. We reason that this decrease in the maximal inter-organelle distance could act to enhance the transport of Ca^2+^ ions from the ER to mitochondria.

In summary, by using an artificial linker to increase contacts between mitochondria and the ER, we uncovered how increased contacts between these two organelles can remodel signalling pathways with consequences for lifespan and disease outcomes.

## MATERIALS AND METHODS

### Genetics and *Drosophila* strains

Fly stocks and crosses were maintained on standard cornmeal agar media at 25°C. The linker (a gift from Dr G. Csordás, Thomas Jefferson University, Philadelphia, USA), which encoded a monomeric red fluorescent protein (mRFP) preceded by the outer mitochondrial membrane targeting sequence mAKAP1 and followed by the ER targeting sequence yUBC6. This construct was codon optimised by site directed mutagenesis and cloned into the pUASTattB vector for PhiC31-mediated site-directed transgenesis. Transgenic flies were generated at the Cambridge fly facility, Department of Genetics, University of Cambridge, Cambridge, UK. *da*GAL4, *elav-*GAL4 and UASmitoGFP were obtained from the Bloomington Stock Centre, UAS*-Αβ_42_ arc* was a kind gift from D. C. Crowther, Department of Genetics, University of Cambridge, Cambridge, UK, and UASmitycam was a gift from Professor Julian Dow, Institute of Molecular Cell & System Biology, University of Glasgow, Glasgow, UK. All experiments on adult flies were performed with males.

### Citrate synthase assay

Citrate synthase activity was measured using the Citrate Synthase Assay kit (Sigma-Aldrich, CS070) as described previously (Garrido-Maraver et al., 2019). The units of citrate synthase were normalised to the protein concentration (mg/ml).

### OXPHOS activities

The activities of NADH: cytochrome c reductase (complex I+III) and succinate: cytochrome c reductase (complex II+III) were determined in fly extracts by spectrophotometry as previously described (Rustin et al., 1994; Spinazzi et al., 2012) and normalized to the protein concentration (mg/ml).

### ATP levels

Five male flies (3- to 5-day-old) were homogenised in 100 μl of 6 M guanidine-HCl in extraction buffer (100 mM Tris and 4 mM EDTA, pH 7.8) to inhibit ATPases. Homogenised samples were subjected to rapid freezing in liquid nitrogen followed by boiling for 5 min. The samples were then cleared by centrifugation and the supernatant was diluted (1/50) with extraction buffer and mixed with the luminescent solution (CellTiter-Glo Luminescent Cell Viability Assay, Promega). The luminescence was measured on an Infinite M200PRO plate reader (TECAN, Switzerland). The relative adenosine triphosphate (ATP) levels were calculated by dividing the luminescence by the total protein concentration, which was determined by the Bradford method.

### Electron microscopy

For transmission electron microscopy, adult fly brains were fixed overnight in 0.1 M sodium cacodylate buffer, pH 7.4, containing 2% paraformaldehyde, 2.5% glutaraldehyde and 0.1% Tween-20. Then, the samples were post-fixed for 1 h at room temperature in a solution containing 1% osmium tetroxide and 1% potassium ferrocyanide. After fixation, the samples were stained with 5% aqueous uranyl acetate overnight at room temperature; then, they were dehydrated via a series of ethanol washes and embedded in TAAB epoxy resin (TAAB Laboratories Equipment Ltd., Aldermaston, UK). Semi-thin sections were stained with toluidine blue, and areas of the sections were selected for ultramicrotomy. Ultrathin sections were stained with lead citrate and imaged using a MegaView 3 digital camera and iTEM software (Olympus Soft Imaging Solutions GmbH, Münster, Germany) with a Jeol 100-CXII electron microscope (Jeol UK Ltd., Welwyn Garden City, UK).

### Larval crawling assay

For crawling analysis, ten larvae were placed on a 92-mm square petri dish with 2% agar and monitored for 10 min under constant lighting and temperature. Video files were converted to images (1 frame per second) and analysed using ImageJ software. The images were imported as stacks and background-subtracted (average of all pictures). The TrackMate plugin (Tinevez et al., 2017) was used to analyse the movement of the larvae using displacement, speed and location parameters. Tracks lasting more than a minute were selected and used for data analysis.

### Microarray acquisition and analysis

RNA was prepared from male adult flies (16 samples in total, four replicates for each genotype). The RNA quality was confirmed using an Agilent 2100 Bioanalyzer (Agilent Technologies, CA, USA). Detailed experimental protocols and raw data were deposited in ArrayExpress under accession E-MTAB-8468. Bioinformatic data analysis was performed using R and Cytoscape. The analysis workflow is available in GitHub (https://m1gus.github.io/Garrido_Maraver_MERCS/).

### Lifespan analysis

Groups of 15 newly eclosed males of each genotype were placed into separate vials with food and maintained at 25°C. The flies were transferred to vials containing fresh food every 2–3 days, and the number of dead flies was recorded.

### Locomotor assay

Locomotor activity was recorded using the *Drosophila* Activity Monitoring System (TriKinetics, Waltham, MA, USA). In each assay, 32 1-day-old male flies of each genotype were grown under a light/dark 12 h:12 h cycle at 25°C. The number of average midline crossings per day were plotted for each genotype and analysed using GraphPad Prism. All experiments were performed in triplicate.

### Climbing performance

Climbing assays were performed using a counter-current apparatus equipped with six chambers. A total of 15–20 3-day-old male flies were placed into the first chamber, tapped to the bottom, and then given 20 s to climb a distance of 10 cm. The flies that successfully climbed 10 cm or beyond within 20 s were then shifted to a new chamber, and both sets of flies were given another opportunity to climb the 10-cm distance. This procedure was repeated a total of five times. After five trials, the number of flies in each chamber was counted. A video demonstrating this technique can be found at https://youtu.be/vmR6s_WAXgc. The climbing index was measured using a weighted average approach with the following formula:
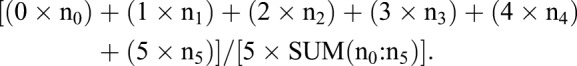
In this formula, *n_0_* corresponds to the number of flies that failed the first trial, and *n_1_* through *n_5_* are the numbers of flies that successfully passed each successive trial. At least 100 flies were used for each genotype tested.

### Mitochondrial calcium imaging

Mitochondrial calcium was monitored by using a fluorescent mitochondria-targeted calcium reporter (mitycam). Mitycam is a modified pericam-based probe with a single excitation wavelength peak at ∼485 nm (Terhzaz et al., 2006). For mitycam imaging, *Drosophila* larval brains were dissected in Schneider's medium. Neurons expressing *UAS-mitycam* and *UAS-linker* driven by *elavGal4* were monitored using a Zeiss LSM510 confocal system. The reporter was excited with an argon 488-nm laser, and the emission signal was filtered through a 505-530-nm bandpass filter. Mitycam signals close to and distant from the linker signal (mRFP) were measured using ImageJ software.

### Measurement of mitochondrial ROS in *Drosophila* adult brain

Adult *Drosophila* brains were dissected in PBS and incubated with 5 μM CellRox Green Reagent, a fluorescent probe for measuring oxidative stress, (Molecular Probes, C10444) for 30 min. After incubation, the brains were washed with PBS for 10 min and imaged on a Zeiss LSM510 confocal microscope. A 100 μm-thick stack was acquired and used to measure the MitoSOX signal using ImageJ software.

### Statistical analyses

Statistical analyses were performed using GraphPad Prism 8 (www.graphpad.com). The data are presented as the mean values, and the error bars indicate±s.d. The number of biological replicates per experimental variable (*n*) is indicated in either the respective figure or figure legend. Significance is indicated as * for *P*<0.05, ** for *P*<0.01, *** for *P*<0.001, and **** for *P*<0.0001. In the violin plots, the solid white line represents the median and the dashed white lines define the interquartile range (the difference between the 75th and the 25th percentile).
